# Systemically and cutaneously distributed ectoparasiticides: a review of the efficacy against ticks and fleas on dogs

**DOI:** 10.1186/s13071-016-1719-7

**Published:** 2016-08-08

**Authors:** Kurt Pfister, Rob Armstrong

**Affiliations:** 1 Parasite Consulting GmbH, Wendschatzstrasse 8, CH-3006 Berne, Switzerland; 2 MSD Animal Health, 2 Giralda Farms, Madison, NJ 07940 USA

**Keywords:** Ectoparasiticide, Acaricide, Insecticide, Permethrin, Fluralaner, Dog

## Abstract

Acaricidal (tick) and insecticidal (flea) efficacy of systemically and cutaneously distributed ectoparasiticide products for dogs are compared based on permethrin and fluralaner as representative molecules. Results of efficacy studies against fleas and ticks are reviewed that show generally good to excellent results. Both externally and systemically distributed treatments have benefits and weaknesses in potentially preventing pathogen transmission by these arthropod vectors.

Four general properties are considered related to the goal of providing optimal reduction in the risk of vector-borne pathogen transmission. These are:

1. Owner adherence to the recommended treatment protocol;
2. Rapid onset of activity following administration;
3. Uniform efficacy over all areas of the treated dog at risk for parasite attachment;
4. Maintenance of high efficacy throughout the retreatment interval.

In considering these four factors, a systemically distributed acaricide can offer an option that is at least as effective as a cutaneously administered acaricide with regard to the overall goal of reducing the risk of vector-borne pathogen transmission.

## Background

Over the last 25 years, considerable advances have been made in the discovery and development of ectoparasiticide products for dogs. This has resulted in an enormous increase in the number of available products to either treat dogs against ectoparasites or to prevent development of ectoparasite populations in the household environment. One consequence of this development is an on-going debate regarding the relative merits of using either an ectoparasite control medicine that is distributed cutaneously on the dog skin surface or an ectoparasiticide that is distributed systemically by the dog’s blood circulation. This discussion regarding active ingredient distribution is independent of questions regarding the mode of administration, because systemically distributed active ingredients may be administered orally, on the skin surface, or by injection. On the other hand, all currently available cutaneously distributed products are exclusively administered externally, on the skin surface.

The on-going debate has recently changed with the commercial introduction of isoxazoline-class ectoparasiticidal medicines. This new class offers systemic, prolonged and highly specific efficacy against multiple genera and species of ectoparasitic arthropods and delivers highly successful control compared to earlier compounds. There are presently three medicines in this class that have received approval for use on dogs: fluralaner (Bravecto, MSD Animal Health, Madison, USA), afoxolaner (NexGard, Merial, Lyon, France) and sarolaner (Simparica, Zoetis, Florham Park, USA). A notable difference between the three currently approved commercial isoxazoline formulations is that fluralaner offers a duration of activity following a single dose that is nearly three times longer compared with the other two [1–3].

The aim of this review is to reconsider the debate between (i) the efficacy against ectoparasites and (ii) the vector-borne disease control of systemically and cutaneously distributed ectoparasiticides in light of the introduction of the isoxazolines. To focus the discussion, systemic and cutaneously distributed products are compared by using a representative example medicine for each class. Fluralaner has been chosen to represent the systemically distributed class and the synthetic pyrethroid permethrin has been chosen as the comparison compound selected for the cutaneously distributed medicine. This review of published data regarding the use of both ectoparasiticides is intended to help veterinary parasitologists and practicing veterinarians when considering their recommendations for effective ectoparasite control in dogs.

### Background on ectoparasite medications for dogs

It has long been known that certain plants, flowers, and roots - or oils and resins extracted from them - have measurable effects against ectoparasites [4–9]. Such extracts include pyrethrins, rotenone and others - and purified active ingredients from these extracts have been commercialised as ectoparasiticides for use on domestic animals. The pyrethrins include various natural insecticidal esters derived from extracts of the pyrethrum plant *Chrysanthemum cinerariifolium* and related plant species. Members of this class have been shown to have “knock-down” properties for arthropods and a low toxicity for mammals. “Knock down” means that treatment leads to a relatively quick parasite drop off after the parasite shows initial hyper-excitation, disorientation and repellence that can be followed by, at a sufficient dose, arthropod death [6, 9]. These properties led to interest in further understanding and testing of these ectoparasitic substances, leading to discovery of the synthetic pyrethroids. These include a range of synthesized compounds developed from the original pyrethrin moiety that are now widely used in various formulations and in a variety of combinations with other substances for the control of ectoparasites of dogs, other domestic animals and people.

In contrast to the plant origin of pyrethrins, isoxazoline class medicines were discovered through searches using “libraries” of potential candidate molecules. The candidates were analysed in batteries of sensitivity tests looking for evidence of potent insecticidal and acaricidal activity based on standardized in vitro and in vivo models. Promising molecules were then evaluated for mammalian safety before proceeding to clinical testing for potential commercial introduction. The commercially available isoxazoline molecules differ in their specific chemical structure, but all are based on the central characteristic isoxazoline moiety.

### A brief discussion of terms used in discussing ectoparasiticide efficacy

Comparative discussions of the properties of antiparasitic compounds involve the introduction of terms that can sometimes be confusing. The most common source reference for these definitions is the World Association for the Advancement of Veterinary Parasitology (WAAVP) guidelines for companion animal ectoparasiticides and these guidelines are used here in attempting to reduce the confusion [10]. **Efficacy** refers to the actual therapeutic response produced by a product against an ectoparasite as determined in a number of controlled studies using infested animals. **Immediate** or **curative** efficacy means the therapeutic effect of a product on a resident ectoparasite population within a defined period from treatment within 24 – 48 h after initial treatment or re-infestation. **Persistent (residual) efficacy** means the extended therapeutic activity of a product measured in days or weeks (normally at time points more than 48 h following the initial treatment).

**Repellency** refers to the action of a product that causes ectoparasites to avoid or leave the dog, or to fail to feed on the dog, i.e. the ability of the compound to prevent the parasite from attaching or migrating onto the dog. A similar concept is the “anti-feeding” effect, which refers to the ability of the compound to stop the parasite from taking a meal from the dog. Three additional concepts that should be clarified are “speed of kill”, “onset of activity” and “onset of effect”. **Speed of kill** refers to the time after treatment for a product to kill a stated percentage of the parasite population. Speed of kill tends to decrease with time following treatment and therefore should be discussed in reference to the length of time elapsed after treatment of the animal. Additionally, the length of time the parasite is exposed to the treated animal is important in interpreting speed of kill data. **Onset of activity** refers to the time taken following first treatment of the animal before an initial killing effect can be measured against parasites already on the host at the time of treatment. **Onset of effect** refers to the time taken for the proportion of parasites killed to reach a previously specified regulatory efficacy level (in Europe this is usually 90 % for ticks and 95 % for fleas) throughout the recommended treatment interval.

There are generally two calculation methods used to summarize efficacy data for statistical comparison of experimental results between groups of test animals. The **geometric mean** - a type of average indicating the central tendency of a set of numbers - is defined as the *n*th root of the product of *n* numbers, while the **arithmetic mean** is defined as the sum of *n* numbers divided by *n.* The arithmetic mean is preferred by registration agencies, possibly because the calculated result tends to be more conservative in estimating efficacy. The geometric mean is less influenced by outlier numbers and therefore this measure is sometimes preferred in scientific reports evaluating ectoparasiticide efficacy. Parasite population numbers have a tendency to increase dramatically if a treatment is ineffective and the geometric mean calculation is less affected in this case. Results of studies that are calculated using the arithmetic mean cannot be reasonably compared to results calculated using the geometric mean - unless the result is either 100 % or 0 %, when the two calculated means are the same.

### Cutaneously distributed ectoparasiticides - using permethrin as an example

#### Short description of permethrin

Permethrin, discovered in 1973 [4], is a synthetic structural derivative of the naturally occurring pyrethrins and is classified as a Type-I pyrethroid because it lacks an ά-cyano group. In veterinary medicine, permethrin is widely used on dogs and various food-producing animal species as a topically administered and cutaneously distributed medication for control and prevention of arthropod parasites [8, 9]. In dogs permethrin is predominantly administered as a spot-on formulation but has also been formulated in collars [11, 12]. Permethrin is also used to impregnate clothes and nets to protect people from flying arthropods, an indication that is particularly valuable for reducing the risk of malaria transmission in tropical and subtropical areas [13].

Permethrin is poorly soluble in water, non-volatile and stable under the effects of light, air, acidity, alkalinity and moisture. These properties contribute to a much greater residual capability of permethrin compared with natural pyrethrins. The lipophilic properties of permethrin provide it with an increased attraction to lipid-containing tissues where it is rapidly metabolised through enzymatic hydrolysis, oxidation and other conjugation processes. This rapid metabolism contributes to the safety of permethrin in dogs. However, cats - unlike dogs - lack the enzyme glucuronyl transferase for conjugating permethrin metabolites and cannot metabolise permethrin at the dosages in dog formulations. Consequently, any contact between cats and products containing permethrin, including contact with permethrin-treated dogs, must be avoided. Clinical signs of toxicity in cats include hypersalivation, muscle tremors, cramps, hyperthermia, motility disorders and lameness and can lead to death if the cat is not rapidly treated [7–9].

### Administration of permethrin or permethrin-containing formulations on dogs

Permethrin formulations for dogs are often mixed with other compounds to improve the insecticidal activity of the combination. Examples are: indoxacarb (Activyl, MSD Animal Health, Madison, USA); fipronil (Frontline Tri-Act/Frontect, Merial, Lyon, France) and imidacloprid (Advantix, Bayer Animal Health, Leverkusen, Germany). Some permethrin combinations for dogs also contain an Insect Growth Regulator (IGR), e.g. permethrin, dinotefuran and pyripoxyfen (abbreviated as DPP) (Vectra 3D, Ceva Santé Animale, Libourne, France). These formulations, whether a single active or a combination, are typically administered directly onto the skin (“spot-on”) between the shoulder blades or on the back after appropriately parting the hair. There is limited transdermal absorption, less than 2 % through mammalian skin, after administration [8, 13]. The lipophilic nature of permethrin means that it is almost insoluble in water [14] and therefore topical formulations can have an oily consistency from the use of appropriate solvents.

### Mode of action on arthropods

Permethrin works after contact with the arthropod and absorption into the arthropod either directly through the outer cuticle or through ingestion during feeding on the host. The lipophilic properties of permethrin lead to distribution along the arthropod nervous system. Permethrin interferes with the voltage-gated sodium channels of neurons by slowing down the activation and inactivation process of the sodium channel gates and significantly prolonging sodium ion influx. This results in continuous nerve discharges causing restlessness, incoordination, tremor, paralysis and respiratory failure and eventually arthropod death [8, 9, 15].

Pyrethroids are much more potent to arthropods than mammals (other than cats) as seen by comparing the difference in comparative lethal doses (LD_50_). The relative potency of permethrin comparing its toxicity for insects with its toxicity to rats is approximately 1400× higher [5], thus underlining the high selective killing potential for arthropods. Additionally, at lower temperatures the potency of pyrethroids increases, adding to the selectivity for poikilothermic insects. The much slower metabolism of permethrin by ester hydrolysis and various oxidase functions in arthropods compared with mammals further increases the selective toxicity for arthropods over mammals, with the notable exception of the cat as described earlier.

### Permethrin distribution on dogs

Permethrin and permethrin-containing spot-on formulations are designed in a way that the active ingredients will spread cutaneously on the dog’s skin surface by local tissue-dependent migration through continuity from the point of administration through the hair coat along the *stratum corneum* with the objective of eventually covering the entire body surface. Experiments specifically undertaken in a small number of dogs to demonstrate this covering objective under specified laboratory conditions have shown that most - but not necessarily all - parts of the dog’s surface had been covered to the same extent and concentration [16, 17]. The lipophilic permethrin is detectable in the hair coat and *stratum corneum* after application, but cannot pass through the *stratum corneum,* nor through the rather hydrophilic dermis [17, 18]. Permethrin is typically applied - according to body weight - in either one or two spots on the dorsal back of the dog and it is possible under normal home treatment conditions that more distant regions of the body may not receive the same coverage as places closer to the application site. Some distant body parts (distal parts of legs, parts of the belly) were not fully covered by permethrin migration in one study [16]. An experimental study on body distribution of permethrin after topical application using six dogs [17] revealed markedly lower and highly variable permethrin concentrations in the *stratum corneum* of the hind legs compared with an area on the dorsal back of the dog, about 10 cm from the drug application site, starting from the first day after treatment. Also, some hair samples taken on day 14 and day 28 after treatment from the hind legs of dogs in this study showed significantly lower permethrin values than hair samples from the back. Additionally, overall permethrin concentrations decreased faster over the 28 day study period in both the hair and the *stratum corneum* of the hind legs compared with the back. These findings suggest that factors affecting the dog’s coat structure and physiology - such as continuous *stratum corneum* desquamation, grooming behaviour and environmental aspects (sun exposure, bathing and shampooing, swimming) could all contribute to a varying degree of superficial dermal abrasion and permethrin removal.

There could be an impact of constant hair coat and skin changes (e.g. *stratum corneum* desquamation) and dog activity (e.g. frequent wetting of the feet and lower limbs during outdoor activity) on the speed and extent of superficial permethrin migration. Thus, the efficacy of topically administered and distributed permethrin products could be reduced in areas distant from the application site. Field trial results in privately owned dogs [19] showed that the highest (surviving) immature tick counts on day 28 after imidacloprid 10 %/permethrin 50 % treatment were seen on the legs. This observation underlined the potential for a greater comparative rate of loss of active ingredient from areas such as the legs. Shampoo bathing is known to effectively remove permethrin from the coat, and thus is an important part of the recommended treatment protocol for exposed cats. Therefore, it is reasonable to believe that routine bathing and swimming in the normal life of a household dog will influence the duration of effect of permethrin subsequent to topical application. These types of effects could result in diminished efficacy and could help to explain the considerable variation in published permethrin efficacy data, particularly towards week 4 after treatment.

### Efficacy of permethrin against ticks

The tick killing effect within 24–48 h after treatment or re-infestation is sometimes called “immediate” or “curative” efficacy, although other terms are also used such as “knock-down” effect [10, 20]. The rapid cuticular penetration and high drug accumulation of permethrin in arthropod tissue [13, 21] generally contribute to a rapid killing efficacy [22]. Therefore, permethrin application usually provides a strong immediate killing effect after initial arthropod exposure.

**Fig. 1:**
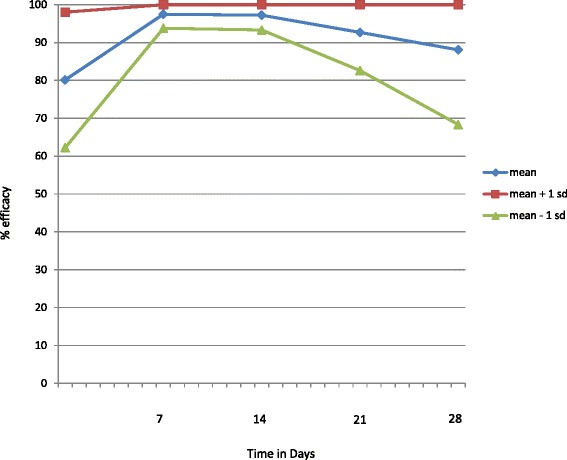
A summary analysis of all 16 published permethrin efficacy studies [16, 23–33] meeting quality and duration standards against ticks (genera are *Ixodes*, *Rhipicephalus*, *Dermacentor* and *Amblyomma*) on healthy dogs to illustrate the range of potential efficacies expected in field use

Permethrin and permethrin-combination products have proven to be effective against various tick species on dogs, following either artificial or natural infestations. Multiple efficacy studies are described in this review, rather than tabulated, because the use of differing methodologies and terminology in the studies would make interpretation of a table very complex. Instead, a simplified graphic representation is used to pull together data from all 16 published studies that provide challenge data meeting basic standards of quality and duration (Fig. 1; see the Discussion section of this paper for further explanation) [16, 23–33]. Efficacy of a topical 65 % permethrin - formulation (Defend Exspot, MSD Animal Health, Madison, USA) on day 1 after treatment was between 75 % (against *Dermacentor variabilis*) and 100 % (against *Ixodes dammini* – now renamed *Ixodes scapularis*) in small (< 15 kg) dogs using a “lower dosage” and was 100 % in heavier (> 15 kg) dogs using a “higher dosage” [16]. Efficacies against *Rhipicephalus sanguineus* (*sensu lato*) on day 3 after topical administration of the same product were 70 % (small dogs) and 49 % (heavier dogs) compared to 96 and 90 %, respectively on day 7 post-treatment. The same 65 % permethrin formulation was used in two other trials [23, 24] and demonstrated efficacies of 96.3 % on day 2 and > 99 % on day 7 after treatment when dogs were exposed for 2 h to adult *Ixodes ricinus*. Permethrin killed adult *Dermacentor reticulatus* ticks and also killed 100 % of unfed nymphs and larvae within 48 h after topical administration and also impaired the embryonic development of eggs from exposed adult female ticks [34, 35].

Many efficacy trials have been conducted with combinations of permethrin and other active ingredients. A combination of imidacloprid 10 %/permethrin 50 % spot-on (Advantix, Bayer Animal Health, Leverkusen, Germany) demonstrated a “curative” efficacy of 74 % against experimental adult *R. sanguineus* (*s.l.*) and of 67 % against *I. ricinus* infestations at 2 days post-treatment [25]. The 48 h post-treatment efficacy of the DPP topical combination (dinotefuran 4.95 %/permethrin 36.08 %/pyriproxifen 0.44 %) (Vectra 3D, Ceva Santé Animale, Libourne, France) was 57.1 % and the efficacy of the combination imidacloprid 8.8 %/permethrin 44.0 % (K 9 Advantix, Bayer Animal Health, Leverkusen, Germany) was 54.3 %, against adult *R. sanguineus* (*s.l.*) The 24 h post-treatment efficacy was only 11.9 % for DPP and 16.7 % for imidacloprid/permethrin [26]. These low efficacies were thought to be associated with ticks that were already attached at the time of treatment and that therefore received comparatively less contact with the active ingredient because they did not need to find an attachment site. However, by day 8 post-treatment, the efficacy of both treatments increased to > 99 % at 24 and 48 h post-challenge. In another study, the same DPP formulation (permethrin 36.08 %, Vectra 3D, Ceva Santé Animale, Libourne, France) had an efficacy of 79.9 % against *R. sanguineus* (*s.l.*) on dogs in a laboratory challenge at 48 h after spot-on treatment application [27].

Field studies with an 8.8 % imidacloprid/44.0 % permethrin formulation (K9 Advantix, Bayer Animal Health, Leverkusen, Germany) found that the 48 h post-treatment efficacy was 93.5 % against adult *Amblyomma americanum* and 88.54 % against adult *R. sanguineus* (*s.l.*) ticks [28]. Significant acaricidal effects (compared with untreated controls) of spot-on permethrin or permethrin-combinations were measured at 6 h after treatment against adult *D. reticulatus*, but higher efficacy rates were not observed until 24–48 h post-treatment. A different approach of calculation was used in this study and the exact efficacy data are not specified [36].

A combination of 50.48 % permethrin and 6.76 % fipronil (Frontline Tri-Act/Frontect, Merial, Lyon, France) in a laboratory challenge had efficacy of 94.4 % and 100 % against *R. sanguineus*, 100 % against *I. ricinus*, and > 99 % against adult *D. reticulatus* two days after treatment [29]. Similarly, 98 % efficacy of a 54.5 % permethrin/6.1 % fipronil combination (Effitix, Virbac, Carros, France) was demonstrated against experimental adult *I. ricinus* infestation two days post-treatment [30]. The contribution of permethrin to these results cannot be quantified given the known acaricidal activity of fipronil.

The above studies show that acaricidal efficacy varies considerably following permethrin administration to dogs. The varying efficacy rates observed between different tick species do not apparently correlate with the concentration of permethrin in the applied formulation. Several studies also show that acaricidal efficacy of permethrin can be weak within the first 48 h after administration, and this was observed in studies with both mono-substance formulations and in combinations with active ingredients including imidacloprid or dinotefuran/pyriproxifen.

A laboratory challenge study observed 100 % efficacy against adult *R. sanguineus* (*s.l.*) and *D. variabilis* after 3 h exposure on day 3 following administration of a combination formulation containing permethrin 44 %/imidacloprid 8.8 %. This study also reported efficacy of only 88.6 % against adult *A. americanum* and 92.9 % against adult *I. scapularis* [31, 32]. However, for the latter tick species, 100 % efficacy was reached after 24 h tick exposure suggesting that timely and extended permethrin contact is essential for increased acaricidal efficacy.

The variety of study designs, tick challenges, efficacy measurement techniques and calculation methods used make study results summaries difficult and confusing. However, the field conditions under which permethrin treatments are used are equally highly variable: for example, the infesting tick species are often not identified and the dose may be inexpertly administered. Therefore, an overall summary of all reported experimental results still provides a valuable indication of the full range of efficacy that might be expected with field administration. To prepare a visual overall summary, mean efficacy was calculated from the 16 published studies with 4 week efficacy results (Fig. 1). In addition to the mean efficacy, an upper and lower bound of one standard deviation above and below is shown indicating the potential efficacy range. Permethrin efficacy peaks between 7 and 14 days post-treatment and it is likely that a portion of ticks infesting treated dogs will survive permethrin treatment during the times outside this peak period. This includes the initial week following first administration and the period of declining efficacy following day 14 after treatment. It is much less likely, but still possible, for ticks to survive during this peak period. The reasons for this efficacy pattern are likely complex and include multiple host, parasite, environment and treatment related factors. One factor is the variation in efficacy against the same tick species isolated from different geographical areas of tick origin: for example, one study reported 92 % efficacy for an imidacloprid/permethrin formulation against a *D. variabilis* isolate from California, whereas the comparative efficacy against an Oklahoma isolate of the same tick species was only 17.5 % [37]. Differences in product formulations and variations in skin surface distribution of the active ingredients can also contribute to differing efficacy results [38]. A comparison of the efficacy of four different permethrin formulations - including a combination with imidacloprid - reported non-significant slight variations [36]. Somewhat lower and more variable efficacy reported on distal body locations of dogs treated with a combination of imidacloprid 10 %/permethrin 50 % (Advantix, Bayer Animal Health, Leverkusen, Germany) support the hypothesis that uneven or partially uneven distribution of active ingredients on the body surface accounts for some efficacy variability [19]. However, further research may be required to better understand the factors underlying reported efficacy variation.

### Long-term (preventive) efficacy against ticks and tick-borne infections

The individual host-seeking patterns, survival strategies and disease epidemiology of the different genera and species of ixodiid ticks ensure that tick control compounds require an extended efficacy period to ensure successful and sustainable control of these ticks. Effective tick control is a prerequisite for reducing the risk of transmission of the associated tick-borne infections. Control does not need to be immediate because there is a grace period between attachment of the tick and transmission of tick-borne organisms [39] during which time effective acaricidal treatment can prevent infectious agent transmission. Additionally, tick-borne infectious agent transmission is more intricate and complex than the simple concept of immediate injection of infectious agents residing in the tick mouth parts. This subject will not be reviewed in this paper; however, it is possible that tick-borne infections may require an on-host stay/feeding/attachment of longer than 24–48 h for successful pathogen transmission [40]. One study showed transmission of *Ehrlichia* more rapidly following tick attachment; however, this study used an intensive laboratory exposure model and the transmission rate did not increase over the initial 24 h of exposure [41].

Most permethrin or permethrin-associated combinations have label indications that offer both immediate and persistent efficacy against ticks over approximately one month following initial treatment. The duration of the preventive efficacy may be 4 weeks, or in some cases even 5 weeks after a single topical administration [16, 19, 29, 36, 42]. However, some label indications may be shorter, for example the 3 week duration of efficacy recommended against *D. reticulatus* for an imidacloprid/permethrin formulation (Advantix, Bayer Animal Health, Leverkusen, Germany) [43].

A single spot-on administration of permethrin (Defend Exspot, MSD Animal Health, Madison, USA) can result in efficacy between 88 and 92 % (against adult *R. sanguineus*) and 86–90 % (against adult *I. dammini* – now *I. scapularis*) as well as between 87 and 99.5 % (against adult *I. ricinus*) for a period of 28 days [16, 23, 24].

A single topical administration of a combination of imidacloprid 8.8 %/permethrin 44.0 % (K9 Advantix, Bayer Animal Health, Leverkusen, Germany) was used in experimental studies, and the following efficacies were measured at 48 h after re-infestation: 97.6 – 91.5 % against adult *R. sanguineus* (*s.l.*) and 100 % – 91.6 % against adult *I. ricinus* over 5 weeks; ≥ 88.8 % against adult *R. sanguineus* (*s.l.*)., ≥ 92 % against adult *D. variabilis*, > 95 % against *A. americanum* and ≥ 96.5 % against *I. scapularis* for a period of 28 days after treatment [25, 32]. In a comparative trial testing DPP against an imidacloprid/permethrin combination for adult *R. sanguineus* s.l. efficacy, the 24 h post-treatment values for imidacloprid 8.8 %/permethrin 44 % on day 29 (91.7 %) were not significantly different from those of the DPP combination (> 96 %) [26]. A field study in Italy confirmed these results with efficacies of 91.6 % against adult *R. sanguineus* (*s.l.*) and > 98 % against a co-challenge with adult and immature *R. sanguineus* s.l. together at 28 days after treatment with a spot-on combination of imidacloprid 10 %/permethrin 50 % (Advantix, Bayer Animal Health, Leverkusen, Germany) [19]. However, on day 7 post-treatment, the acaricidal efficacy against adult *R. sanguineus* (*s.l.*) remained below 90 % before increasing to > 90 % thereafter until day 28 post-treatment.

Lower values of only 81.4 % efficacy against adult *R. sanguineus* (*s.l.*) were detected 30 days after treatment with imidacloprid 10 %/permethrin 50 % (Advantix, Bayer Animal Health, Leverkusen, Germany) [44] and also 24 h post-treatment efficacy < 90 % were measured against adult *D. reticulatus* with the same topically administered product over a 29 day study period [45].

A single topical spot-on administration of the combination 6.76 % fipronil/50.48 % permethrin (Frontline Tri-Act/Frontect, Merial, Lyon, France) to dogs under laboratory conditions, resulted in preventive efficacy of > 96 % against *R. sanguineus* (*s.l.*)., > 99 % against *I. ricinus* and > 99 % against *D. reticulatus* at 48 h following re-infestation over a study period of 28 to 30 days [29, 33]. Similarly, protection against *I. ricinus* at 48 h post-infestation ranged between 93 % on day 30 and 95 % on day 37 after administration following a single spot-on administration of a combination of 6.1 % fipronil/54.5 % permethrin (Effitix, Virbac, Carros, France) [31].

The reported persistent efficacies of permethrin or permethrin-combination products show that permethrin is likely to provide a considerable preventive potential against arthropod transmitted infections. However, efficacies are less than 100 % at most time points following treatment. Therefore, permethrin treatment protocols should be considered to reduce the risk of transmission, but do not completely eliminate this risk. In a field evaluation on the prevention of tick bites in dogs with a 10 % imidacloprid/50 % permethrin combination (Advantix, Bayer Animal Health, Leverkusen, Germany), it was found that treatment application once or twice monthly led to a highly significant 95.57 % reduction of the risk of *Ehrlichia canis* transmission in an endemic area in southern Italy [46]. Continuous treatment of young dogs in a *R. sanguineus* (*s.l.*) - endemic shelter by application of a 10 % imidacloprid/50 % permethrin combination (Advantix, Bayer Animal Health, Leverkusen, Germany) every 21 ± 2 days over one year, produced an overall tick efficacy of 97.9 % [47]. The reductions in incidence density were: 94.6 % (*E. canis*), 94.4 % (*Babesia* spp.) and 81.8 % (*Anaplasma platys*), when treated dogs were compared with untreated control dogs. Post-treatment tick efficacy in another study ranged between 96.1 and 98.9 % for a period of 4 weeks following spot-on administration of imidacloprid 10 %/permethrin 50 % (Advantix, Bayer Animal Health, Leverkusen, Germany) against *E. canis*-infected *R. sanguineus* [48]. *Babesia canis* transmission by *D. reticulatus* ticks was prevented for 28 days following spot-on administration of a 54.5 % permethrin - 6.1 % fipronil combination (Effitix,Virbac, Carros, France) [49].

Other studies have documented similar protective efficacy rates against *Anaplasma phagocytophilum*, *A. platys*, or *Borrelia burgdorferi* following spot-on administration of permethrin or permethrin combinations to dogs [50–52]. In summary, these studies confirm that permethrin treatment can dramatically reduce, but not eliminate the risk of tick-borne pathogen transmission.

### Repellent (mechanism/potential) effect and anti-feeding

Permethrin exerts a potent repellent effect against a variety of arthropods including ticks [23]. This effect may originate from the contact chemoreception channel found on receptor cells in sensilla on the ventral side of the arthropod tarsus [53]. Therefore, the primary repellent activity of pyrethroids would be via contact irritancy rather than the “space-repellency” demonstrated for *Aedes aegypti* [54, 55] now more commonly known as the “anti-feeding” effect. It is not clear whether anti-feeding in ticks is a chemical process directly limiting the feeding behaviour or whether another mechanism is involved.

Repelled ticks may survive more easily than previously thought [31], because only 8.3 % of moribund or apparently dead *A. americanum* ticks at 3 h after a 10 min exposure to imidacloprid/permethrin 21 days after treatment could still be considered dead or moribund 24 h later. Nevertheless, reported repellency results are performed under variable conditions and there is a lack of standardized protocols [56]. The main sources of variability between studies are the length of time of tick exposure to the treated animal and the methods of tick collection following exposure to the treatment [57].

### Repellency (duration)

A single administration of 65 % permethrin spot-on (Defend Exspot, MSD Animal Health, Madison, USA) in laboratory challenge studies led to significant repellent activities against adult *I. ricinus* measured following exposure for 2 h at either 2 or 7 days after treatment and also over a period of at least 4 weeks compared with either untreated controls, fipronil treated dogs or selamectin treated dogs [23, 24]. Although this formulation contains a higher permethrin concentration, the lower volume applied to the dog results in a dose of active ingredient that is equivalent to the dose delivered with lower strength formulations. Experimental challenge trials measured a repellent-like effect (a term used in this study to describe an apparent rapid irritation effect) of 83 % for a single imidacloprid 8.8 %/permethrin 44.0 % (K9 Advantix, Bayer Animal Health, Leverkusen, Germany) spot-on treatment after a 10 min exposure of *R. sanguineus* (*s.l.*) 3 days after treatment that decreased to < 60 % on days 7, 14, 21, 28. The repellent-like effect was between 93 and 63 % for *D. variabilis*; between 86 and 68 % for *A. americanum;* and between 72 and 52 % for *I. scapularis* on these same days [31, 32].

Similar results were reported in a trial evaluating the repellent activity of a fipronil-permethrin combination (Frontline Tri-Act/Frontect, Merial, Lyon, France) against *D. reticulatus*, with repellency rates after a 4 h exposure of 56.5–73.5 %; after 12 h of 76.3–92.9 %; and after 24 h of 83.9–96.5 %. In the same experimental set-up, prevention of attachment ranged between 64.1 and 79.7 % at 4 h post exposure, between 79.1 and 94.2 % at 12 h post-challenge and between 84.2 and 99.6 % at 24 h post-challenge for up to 4 weeks [33].

### Efficacy against fleas

The “knock-down effect” is the most important action of permethrin against insects and this is caused by rapid penetration of permethrin through the arthropod cuticle [21]. This subsequently causes selective inhibition of specific cell functions without immediately killing the insect. Sodium channels, for example, can retain selectivity for sodium ions and conductance after exposure to pyrethroids. As a consequence, following permethrin exposure cells can either function normally in a new state of hyperexcitability or - with exposure to a higher permethrin dose - the cell membrane will depolarize and there will be a conduction block followed by repetitive nerve discharges. This will lead to loss of function and an accelerated speed of kill observed macroscopically as “knock-down” or quick death of the insect [8, 16]. This “knock-down” or “immediate therapeutic effect” should be assessed at either 24 (European Medicines Agency) or 72 h (WAAVP) post-treatment [10, 20] and the “speed of kill” can also be more precisely determined or characterized by additional counts at 4, 8, or 12 h post-treatment exposure.

Of course, successful flea control can only be achieved with “persistent” efficacy throughout an extended period because of the environmental life stages components of the flea life cycle. Relatively few published studies are available on the persistent efficacy of permethrin against fleas as a mono-substance, but there are many studies on permethrin-combinations against fleas and a few on other insects.

Post- treatment *Ctenocephalides felis* efficacy 72 h after Defend Exspot (MSD Animal Health, Madison, USA) application using a dose according to the size of the dog was 93 % for dogs less than 15 kg, and 92 % for dogs over 15 kg [16]. Persistent flea adulticidal efficacy of 99.4 % was seen within 24 h and larvicidal efficacy of 99.2 % against *C. felis* larvae on day 3 after 12 h permethrin exposure (Advantix, Bayer Animal Health, Leverkusen, Germany) [25]. An adulticidal effect against *C. canis* of > 99 % was seen following 48 h exposure to a 4.95 % dinotefuran, 36.08 % permethrin and 0.44 % pyriproxifen (Vectra 3D, Ceva Santé Animale, Libourne, France)) topical treatment [58]. The same combination product killed 12.7 % of *C. felis* within the first 5 min after topical treatment and the average insecticidal efficacy of this combination was 86 % at 1 h and 95.3 % at 4 h after infestation [59]. Dogs treated with a combination of fipronil and permethrin (Frontline Tri-Act/Frontect, Merial, Lyon, France) showed efficacy of >99 % at 6 and 24 h after weekly challenges for up to 1 month [60].

A summary of five separate studies reported that a single topical treatment with a combination of 6.76 % fipronil/50.48 % permethrin (Frontline Tri-Act/Frontect, Merial, Lyon, France) significantly reduced the number of adult *C. felis* between 98.4 and 100 % at 24 and 48 h post-treatment or post-infestation [61]. This level of efficacy cannot be attributed to just the permethrin in the combination, and this immediate efficacy is apparently greater than observed in other studies following treatment with permethrin-containing combinations. Even at this level of efficacy there are still a few fleas surviving treatment with permethrin and permethrin-combination medications.

### Repellent activity against fleas

Flea repellency, unlike tick repellency, is difficult to appropriately define and measure because fleas attack and bite so rapidly after arriving on the host. Adult cat fleas begin feeding almost immediately once they find a host, with many fleas feeding within minutes. In one study, 25–60 % of fleas were blood fed within 5 min and in another study the volume of blood consumed by fleas was quantifiable within 5 min. Feeding is so rapid that partially digested blood can be defecated in as little as 2–6 min after fleas acquire a host [62]. Specific studies on the residual activity against fleas in both the USA and Europe found that, despite appropriate flea treatment using a medicine with both insecticidal and repellent products, up to 92 % of infesting fleas will bite and consume at least some blood before being killed [62, 63]. Therefore the primary objective of flea treatment is to rapidly reduce flea numbers leading to elimination of the population in the household, rather than to prevent newly arriving fleas from taking an immediate bite.

### Persistent efficacy against fleas

Treatment with permethrin alone (Defend Exspot, MSD Animal Health, Madison, USA) achieved a *C. felis* efficacy at 72 h post-treatment of 93 % for dogs < 15 kg and 92 % for dogs > 15 kg. The efficacy for both groups of dogs was > 93 % at all time points during the 4 weeks [16]. It is likely that treatment of dogs with permethrin alone does not achieve the efficacy level required for effective persistent flea control. Just as with tick efficacy studies, there are many more studies that measure persistent flea efficacy with permethrin combination products than with permethrin alone. However, the interpretation of these flea studies is more complex because combination ingredients are included for their potent insecticidal or insect growth regulatory activity. Therefore, these study results are not summarized here because it is impossible to know how much of the activity should be attributed to permethrin rather than the other active ingredients.

### Ectoparasite control via a systemically distributed drug using fluralaner as an example

#### Short description of fluralaner

Fluralaner is a novel, recently developed systemically distributed molecule of the isoxazoline class with a highly selective ectoparasiticidal activity achieved through blocking arthropod γ-aminobenzoic acid (GABA) and glutamate-ligand gated chloride channels [64–67]. It is a non-competitive GABA-receptor antagonist with a high selective toxicity for arthropod neurons over mammalian neurons [67–69]. In vitro studies have indicated that fluralaner exhibits a many fold better arthropod specific GABA-gated chloride channel inhibition than fipronil [65]. By contrast, fluralaner blocking activity on rat GABA receptors was very weak and a fluralaner dose more than 5× the maximum recommended oral clinical dose was well tolerated by dogs [70]. Fluralaner is well tolerated by young dogs (≥ 8 weeks) and by collies carrying a deletion mutation of the Multi-Drug-Resistance gene [70, 71]. Fluralaner is not toxic for cats and is commercially available as a topical formulation for use on this species.

Fluralaner (Bravecto, MSD Animal Health, Madison, USA) is rapidly absorbed after oral administration and reaches maximum plasma concentrations on average within 24 h and is quantifiable in plasma for up to 112 days after a single oral administration [67]. Absorption is slightly slower following topical administration [72] and the depletion curve may be slightly longer. Fluralaner has a relatively high apparent volume of body distribution and a low clearance followed by a long elimination half-life. These properties account for the prolonged activity of this compound against ticks and fleas after a single oral dose [67]. Elimination is primarily hepatic with essentially no detection of excretion through renal filtration [67].

### Fluralaner administration to dogs

Fluralaner is presented as either a palatable chewable tablet (Bravecto Chew, MSD Animal Health, Madison, USA) or as a topical solution (Bravecto Spot-On, MSD Animal Health, Madison, USA), with both presentations dosed according to the weight of the dog. Gastrointestinal absorption after oral administration is sufficiently rapid that onset of activity against fleas can be detected at 2 h following oral administration [67, 73].

The area under the curve (AUC) and maximum plasma concentrations (Cmax) were increased in dogs fed at the time of treatment compared with dogs fasted for the previous day, indicating that administration at the time of feeding increases bioavailability of the oral formulation (Bravecto, MSD Animal Health, Madison, USA) [74], although this is not a factor affecting systemic bioavailability after topical administration. Concurrent treatment with either milbemycin - praziquantel, a deltamethrin collar or therapeutic doses of ivermectin were well tolerated by treated dogs and did not lead to observed treatment associated adverse reactions [75–77].

### Mechanism of action of fluralaner

Isoxazolines block the ligand-gated chloride channels of both GABA and glutamate receptors [64, 67, 69]. Generally GABA receptors form cation channels while glutamate receptors form anion channels and both are critically important for effective invertebrate neurotransmission. The isoxazoline mode of action exhibits a unique and selective inhibitory action of invertebrate GABA and glutamate-gated chloride channels compared with activity in mammalian neurons [65]. Anion (inhibitory) glutamate channels are only found in invertebrates, so this activity further increases the arthropod specificity of fluralaner. In the invertebrate neurotransmission system, each ligand-gated chloride (anion) channel mediates a fast inhibitory synaptic transmission through the enhancement of chloride ion permeability through the postsynaptic membrane, which subsequently leads to paralysis and death of fleas and ticks and other arthropods [65, 78].

The potential role of GABA receptors as an insecticidal target was first shown in the 1980’s with the organochlorines dieldrin and lindane [64, 79, 80]. However, this opportunity for novel insecticidal or acaricidal activity was not further developed until years later with the discovery and commercial introduction of the phenylpyrazoles (e.g. fipronil) formulated for topical administration only and also distributed cutaneously [64]. However, fluralaner shows a many fold higher receptor binding on arthropod GABA-gated chloride channels than phenylpyrazoles [65].

### Mode of action of fluralaner in dogs

After oral administration and absorption in the digestive tract or topical administration and transdermal absorption, fluralaner is rapidly distributed by the circulatory system and maximum plasma levels are on average reached within 24 h after oral or approximately 7 days after topical administration in dogs. The systemic distribution is quantitatively detectable in plasma (> 10 ng/ml) for 112 days post-treatment following oral administration, thereby reflecting a long systemic persistence and a slow elimination process in the dog, both apparently independent of dose [67, 72]. Furthermore, a high apparent volume of distribution of fluralaner in tissues and a low clearance rate [67] further contribute to the long-lasting systemic availability of fluralaner in the dog. This persistent availability has proved to deliver flea and tick killing efficacy for at least 12 weeks after a single oral or topical administration [1, 81, 82].

Fluralaner delivers a potent ectoparasiticidal effect following ingestion by the parasite. Therefore, exposure to the active ingredient occurs when the arthropod bites the host and feeds on subcutaneous tissue fluids and blood. The systemic distribution of fluralaner means that there is no potential interference or other negative impact on post-treatment efficacy associated with environmental pressures (including bathing, shampooing, sun, rain, etc.) that can potentially occur with cutaneously distributed ectoparasiticides. Additionally, cross-contamination with other household pets is not a concern. Topical administration of fluralaner (Bravecto Spot-On, MSD Animal Health, Madison, USA) has been proved to be associated with no influence on flea and tick efficacy in dogs that are either immersed in water or shampooed between 3 and 84 days after administration [83]. No data are published on dogs immersed or shampooed before 3 days, although efficacy was already 100 % against fleas and ticks (*I. ricinus*) at the 3 day test suggesting that the likely time of impact is well before this time point [83].

### Acaricidal efficacy of fluralaner

#### Immediate efficacy

Immediate efficacy results are important for eliminating existing tick infestations to reduce owner concerns regarding observation of tick infestations on their dogs and for reducing the risk of transmission of tick-borne infections [12]. A series of field and laboratory studies demonstrated that a single dose of fluralaner administered to dogs as a chewable tablet is highly efficacious against common and important ectoparasitic Ixodiid ticks infesting dogs including *I. ricinus, I. scapularis, D. reticulatus, D. variabilis*, *R. sanguineus* and *A americanum* [1, 81, 82]. In addition, following oral fluralaner administration, efficacy was demonstrated against the Australian paralysis tick *Ixodes holocyclus* [84].

The rapid systemic distribution of fluralaner leads to a targeted action on ticks through subcutaneous tissue fluids and blood. Therefore, the tick is exposed to a small but very potent dose of fluralaner on initial feeding and is rapidly killed at an early stage following attachment [85]. Investigations using tick weights and the coxal index as a tick growth parameter showed that *I. ricinus* ticks attached to fluralaner treated dogs did not become engorged [86]. Experimental studies using adult *I. ricinus* have shown tick killing activities of 89.6 % at 4 h, 97.9 % at 8 h, and 100 % at 12 and 24 h. Using tick counts at 8 h after re-infestation, efficacy was 96.8 % at 4 weeks and 83.5 % at 8 weeks post-treatment, whereas the efficacies at 12 and 24 h after re-infestation were at least 98.1 % over the entire 12 week period [87]. A comparative study against *R. sanguineus* and *D. reticulatus* following a 24 h exposure measured efficacy that declined from 100 % at 30 days to 65.7 % at 84 days after a single oral administration [88, 89]. Persistent fluralaner efficacy of 100 % for 115 days when assessed 72 h after treatment was measured against the Australian paralysis tick *I. holocyclus* following a single oral dose of at least 25 mg fluralaner [84]. High fluralaner acaricidal activity was shown following 48 h exposure of immature *R. sanguineus* (*s.l.*) in an in vitro contact study and by artificially feeding *Ornithodorus moubata* nymphs [85]. Efficacy against juvenile tick stages has also been observed under field conditions in Europe suggesting a larger tick control spectrum for fluralaner [81].

### Persistent efficacy of fluralaner against ticks

A single oral administration of fluralaner protected dogs against > 99 % of European ticks including adult *R. sanguineus* (*s.l.*), *I. ricinus*, *I. hexagonus*, *D. reticulatus* and nymphs and larvae of *Ixodes* spp. within 24 h after treatment and for a period of 12 weeks under field conditions [81, 87]. Similar potent tick-killing efficacy (> 98 %) was measured against an experimental adult *I. ricinus* infestation within a 12 to 24 h post-treatment period for a period of 12 weeks after a single oral administration of fluralaner [87]. No other systemic acaricidal treatment is commercially available that provides this duration of protection [87]. The immediate and persistent tick killing efficacy is able to reduce the risk of tick-borne infection transmission. This protective effect was demonstrated in dogs that were administered a single oral fluralaner dose and were then 100 % protected from experimental challenge with *B. canis*-infected *D. reticulatus* ticks on days 2, 28, 56, 70 and 84 after treatment. Exposed but untreated dogs in the 112 days study became infected with *B. canis* demonstrating the validity of the challenge [90].

### Tick repellent activity of fluralaner

Fluralaner is a systemically distributed antiparasitic agent and by definition is not repellent.

### Flea efficacy of fluralaner

In vitro studies [65] found that fluralaner is highly effective against *C. felis, Lucilia cuprina* and *Aedes aegypti* therefore demonstrating its insecticidal efficacy potential. In vivo studies have measured immediate flea efficacy detectable at 1 h post-treatment, with a significant difference from controls at 2 h and reaching between 99.4 and 100 % at 8, 12, 24 and 48 h after a single oral fluralaner treatment [73, 91]. These efficacy results are proof of rapid uptake and distribution of fluralaner following oral administration [86].

### Persistent fluralaner efficacy against fleas

Speed of kill studies with a single oral administration of fluralaner treated dogs confirmed flea efficacy > 91 % at 4 h after re-infestation for 8 weeks after initial treatment and efficacy ≥ 98 % at 8, 12 and 24 h after re-infestation for a period of 12 weeks [73]. Sub-insecticidal blood concentrations (in an in vitro study) of 12.5 ng fluralaner/ml led to a 100 % cessation of flea reproduction [86]. In vivo experimental work following a single oral dose of fluralaner measured 100 % efficacy at 48 h after initial treatment and following repeated *C. felis* infestations over 4 months with a corresponding reduction of flea egg production of 99.9 % [91]. All fleas were killed in less than 24 h on fluralaner treated dogs [73] over a 12 week period after treatment which is well before the expected onset of egg laying. Therefore fluralaner treatment completely prevents renewal of the flea population through egg-laying into the environment as confirmed in a simulated home environment and under experimental conditions [91, 92]. A European multi-center field study [81] demonstrated flea-control efficacies in privately owned dogs of 99.9 % for 12 weeks and 97.39 % of previously flea-infested households were flea-free after this 12 week period. A USA field study in treated dogs found that the mean geometric flea count reduction was ≥ 99.7 % at 4, 8 and 12 weeks following a single oral fluralaner administration [93]. In both of these field studies, alleviation of associated flea-allergic dermatitis (FAD) clinical signs was recorded.

### Fluralaner flea repellent activity

As discussed previously, repellent activity is not thought to be relevant for flea control. Fluralaner is a systemically distributed antiparasitic agent and by definition does not have repellent activity against arthropods.

### Discussion on use of systemically versus cutaneously distributed acaricides for reducing vector-borne infection transmission risks

This review presents published data for both permethrin as an example of a cutaneously distributed ectoparasiticide treatment with proven repellency, and fluralaner as an example of a systemically distributed ectoparasiticide. Both options for ectoparasiticide control bring advantages and disadvantages for ectoparasite treatment, as can be seen from the data presented. In general, the repellent treatment offers the potential to keep the treated animal free of ectoparasites while minimizing (but never eliminating) the risks associated with parasite feeding. However, a tendency towards a slower onset of efficacy; lack of rapid and uniform spread over the animal surface; the potential for loss from the skin surface during the retreatment interval; variable activity against different tick species; and declines in efficacy over the retreatment interval are potential weaknesses for a cutaneously distributed repellent treatment. In contrast, the systemically distributed treatment offers rapid and relatively uniform distribution via blood circulation to all body areas; faster onset of activity (although this could vary depending on the potency of the systemically active and distributed molecules considered); and the potential for a longer duration of action (with the possible exception of cutaneously distributed treatments applied using a slow release collar formulation). However, the flea or tick is specifically targeted by the systemically distributed treatment when it tries to feed. Therefore, it is not possible, based on the available published information, to conclusively say that either treatment approach will be superior to the other in all circumstances. Additionally, there is evidence that different ixodid tick species have variable sensitivities to both types of treatments.

Recognizing that neither treatment distribution method can completely eliminate the risk of tick-borne infection transmission under all circumstances and furthermore that the question of comparative efficacy can never be settled with a single experimental laboratory challenge, what useful recommendations can be drawn from this review? 

First, veterinarians should continue to be careful to counsel dog owners regarding additional actions to take to reduce the risk of tick-borne infection transmission, along with the administration of an effective acaricide. These additional actions are beyond the scope of this review, and can include risk reduction activities such as vaccination, avoiding high risk exposure areas, skin examination, tick removal, and professional health examination.

Secondly, in general, veterinarians could consider four aspects of an acaricide with the goal of providing optimal reduction in the risk of tick-borne disease transmission. These aspects are:

1. Owner adherence to the recommended treatment protocol;
2. Rapid onset of activity following administration;
3. Uniform efficacy over all areas of the treated dog at risk for parasite attachment; and
4. Maintenance of high efficacy throughout the retreatment interval.

Each of these points is considered in more detail below. Adherence refers to the dog owner’s obedience in following the prescribed application instructions for the pet’s medication. Lack of adherence is the most common cause for efficacy failure of ectoparasiticide treatments [94]. Owners may not follow through with prescribed treatment recommendations because they are not seeing ectoparasites at the time when retreatment is due; because they have concerns regarding their own exposure to the treatment; because they are concerned that the treatment could have an impact on their pet; because they think that the risk of renewed ectoparasite infestation is low; because they do not like the cosmetic appearance of an external treatment application site; because their dog dislikes the sensation of treatment application at the site; or, in the case of an oral medication, if the dog is sensitive and regurgitates the medication.

Studies that have surveyed owners for their adherence to cutaneously distributed treatment application instructions have found that owners tend to not follow directions. There are no similar surveys available yet with regard to owner adherence to administration of systemically distributed treatments. If it is assumed that adherence rates are equivalent for both approaches, then it follows that the duration of the persistent efficacy of the product becomes an important factor. Adherence failure only occurs at the time when a retreatment should be administered and a treatment with a longer persistent efficacy will require fewer administrations. It is likely that ease of treatment administration and the acceptance of the treatment by the dog will be additional factors that affect owner adherence to retreatment recommendations. These considerations may differ between individual animals and the veterinarian may want to keep these aspects in mind when considering whether to select an orally or topically administered treatment.

Neither a systemically, nor cutaneously distributed ectoparasiticide will have an inherently more rapid speed of onset of activity. However, a systemically distributed acaricide will have rapid circulation in the blood - and uniform exposure to the ectoparasite in all areas of the body. Therefore, the systemically distributed treatment may be a better choice for this aspect.

**Fig. 2:**
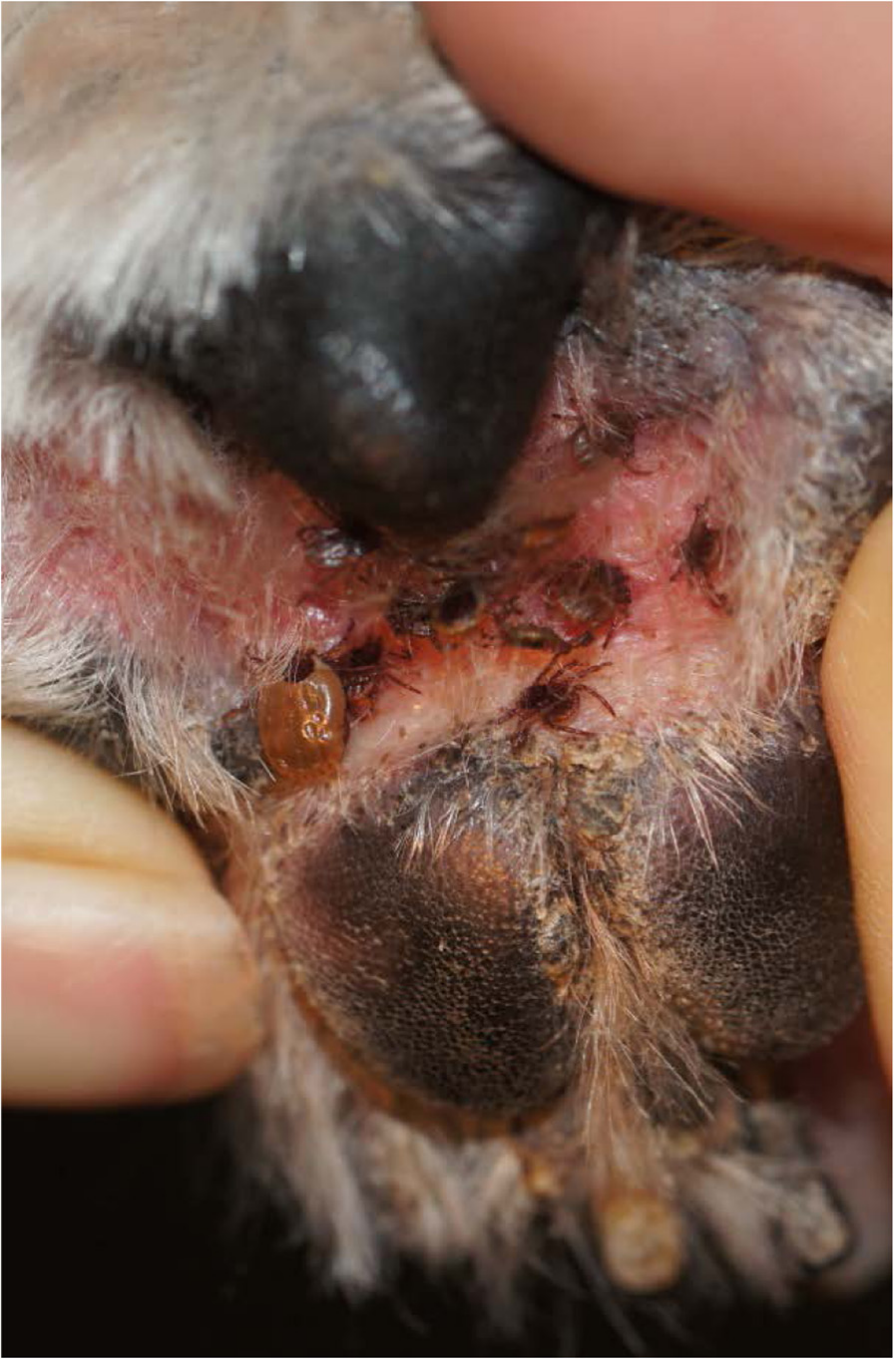
Paw of a dog treated 7 days previously with a permethrin combination product showing multiple attached *Rhipicephalus* sp. ticks. Photo Dr M. Canfield, used with permission

The attached photograph (Fig. 2) shows *Rhipicephalus sanguineus* (*s.l.*) ticks attached between the footpad of a dog that was treated 7 days previously with a permethrin combination product at a time when the treatment should be providing peak efficacy (Fig. 1), and illustrates the difficulties of delivering a cutaneously distributed treatment to the extremities.

Persistent efficacy is very important because of the need to maintain ectoparasiticide control throughout the retreatment interval to reduce the arthropod-borne disease transmission risk. A repellent treatment would offer better reduction of tick-borne infection transmission if it could completely prevent all arthropods from even trying to initiate feeding. Of course, for the flea it is clear that the rapidity of feeding following infestation precludes any benefit for a repellent treatment. However, there is a potential opportunity to repel ticks before feeding, and published efficacy data suggest that there is a “sweet spot” following administration of an externally active repellent product such as permethrin. This “sweet spot” would be the time at which the product has achieved its maximal dispersal over the epidermis before processes such as desquamation, abrasion and environmental wetting have reduced active ingredient levels - initially most likely in the lower legs, axillae, tail and perianal areas. The exact timing of this “sweet spot” is likely to vary and has not been specifically measured, but the overall summary of efficacy results for permethrin (Fig. 1) suggest that it is around 7–14 days following administration. The high level of persistent efficacy observed following administration of fluralaner shows that a systemically distributed treatment can deliver persistent acaricidal efficacy as strong as the comparable efficacy delivered by permethrin treatment during the “sweet spot”. This efficacy can prevent transmission of tick-borne infections such as *B. canis* [90]; however, this may not be true of all infections, or of all systemic distributed acaricides.

## Conclusions

No currently available acaricidal treatment can completely prevent transmission of tick-borne diseases. However, considering the factors of: owner adherence, immediate efficacy, body surface area of protection, persistent efficacy and adherence - a long lasting systemically distributed acaricide can offer an optimal option with regard to reducing tick-borne disease transmission risk. One additional possibility may be to offer combination therapy with both types of active ingredient – a possibility that remains to be evaluated.

## Abbreviatons

AUC, area under the curve; DPP, dinotefuran, permethrin, pyriproxifen; EMA, European Medicines Agency; FAD, flea-allergic dermatitis; GABA, γ aminobenzoic Acid; WAAVP, World Association for the Advancement of Veterinary Parasitology

## References

[1] European Commission. Community register of veterinary medicinal products, Product information, Annex 1 Summary of product characteristics Bravecto. 2014. Available from: URL: ec.europa.eu/health/documents/community-register/html/v158.htm. Accessed 1 June 2016.

[2] European Commission. Community register of veterinary medicinal products, Product information, Annex 1 Summary of product characteristics NexGard. 2015. Available from: URL: ec.europa.eu/health/documents/community-register/html/v159.htm. Accessed 1 June 2016.

[3] European Commission. Community register of veterinary medicinal products, Product information, Annex 1 Summary of product characteristics Simparica. 2016. Available from: URL: ec.europa.eu/health/documents/community-register/html/v191.htm. Accessed 1 June 2016.

[4] Elliott M, Farnham AW, Janes NF, Needham PH, Pulman DA, Stevenson JH (1973). A photostable pyrethroid. Nature.

[5] Elliott M (1976). Properties and applications of pyrethroids. Environ Health Perspect.

[6] Casida JE (1980). Pyrethrum flowers and pyrethroid insecticides. Environ Health Perspect.

[7] Blagburn BL (2003). Permethrin, a type I synthetic pyrethroid: history and properties. Suppl Compend Contin Educ Pract Vet.

[8] Gupta R, Gupta R (2007). Pyrethrins and pyrethroids. Veterinary toxicology, basic and clinical principles.

[9] Löscher W, Ungemach RF, Kroker R (2003). Pharmakotherapie bei Haus- und Nutztieren.

[10] Marchiondo AA, Holdsworth PA, Fourie LJ, Rugg D, Hellmann K, Snyder DE (2013). World Association for the Advancement of Veterinary Parasitology (WAAVP) 2nd edition: Guidelines for evaluating the efficacy of parasiticides for the treatment, prevention and control of flea and tick infestations on dogs and cats. Vet Parasitol.

[11] Schnieder T (2006). Veterinärmedizinische Parasitologie.

[12] Deplazes P, Eckert J, von Samson-Himmelstjerna JEG, Zahner H (2012). Lehrbuch der Parasitologie für die Tiermedizin.

[13] Institute of Medicine (2003). Gulf War and Health: Vol. 2. Insecticides and solvents.

[14] Ferroglio E, Trisciuoglio A, Genchi C (2013). L'evoluzione dei piretroidi come strumento di controllo delle ectoparassitosi. Summa.

[15] Shafer TJ, Meyer DA, Crofton KM (2005). Developmental neurotoxicity of pyrethroid insecticides: critical review and future research needs. Environ Health Perspect.

[16] Lorenz JA, Peters LJ (1994). Defend Exspot, insecticide for dogs: Professional Services Department, Mallinkrodt Veterinary.

[17] Lüssenhop J, Stahl J, Wolken S, Schnieder T, Kietzmann M, Bäumer W (2012). Distribution of permethrin in hair and stratum corneum after topical administration of four different formulations in dogs. J Vet Pharmacol Therapeut.

[18] Bast GE, Kampffmeyer HG (1996). No effect of albumin on the dermal absorption rate of hydrocortisone 21-butyrate, permethrin or diflunisal in the isolated, single-pass perfused rabbit ear. Skin Pharmacol.

[19] Otranto D, Lia RP, Cantacessi C, Galli G, Paradies P, Mallia E (2005). Efficacy of a combination of imidacloprid 10 %/permethrin 50 % versus fipronil 10 %/(S)-methoprene 12 %, against ticks in naturally infected dogs. Vet Parasitol.

[20] European Medicines Agency, Committee for Medicinal Products for Veterinary Use: guideline for the testing and evaluation of the efficacy of antiparasitic substances for the treatment and prevention of tick and flea infestation in dogs and cats. London: EMEA/CVMP/EWP/005/2000-Rev.2; 2007. p. 1–16

[21] Shah PV, Monroe RJ, Guthrie FE (1983). Comparative penetration of insecticides in target and non-target species. Drug Chem Toxicol.

[22] Faulde MK, Uedelhoven WM, Robbins RG (2003). Contact toxicity and residual activity of different permethrin-based fabric impregnation methods for *Aedes aegypti* (Diptera: Culicidae), *Ixodes ricinus* (Acari: Ixodidae), and *Lepisma saccharina* (Thysanura: Lepismatidae). J Med Entomol.

[23] Endris RG, Matthewson MD, Cooke MD, Amodie D (2000). Repellency and efficacy of 65 % permethrin and 9.7 % fipronil against *Ixodes ricinus*. Vet Ther.

[24] Endris RG, Cooke D, Amodie D, Sweeney DL, Katz TL (2002). Repellency and efficacy of 65 % permethrin and selamectin spot-on formulations against *Ixodes ricinus* ticks on dogs. Vet Ther.

[25] Epe C, Coati N, Stanneck D (2003). Efficacy of the compound preparation imidacloprid 10%/permethrin 50% spot-on against ticks (*I. ricinus*, *R. sanguineus*) and fleas (*C. felis*) on dogs. Parasitol Res.

[26] Varloud M, Fourie JJ (2015). One-month comparative efficacy of three topical ectoparasiticides against adult brown dog ticks (*Rhipicephalus sanguineus* s.l.) on mixed-bred dogs in controlled environment. Parasitol Res.

[27] Horak IG, Fourie JJ, Stanneck D (2012). Efficacy of slow-release collar formulations of imidacloprid/flumethrin and deltamethrin and of spot-on formulations of fipronil/(s) - methoprene, dinotefuran/pyriproxyfen/permethrin and (s) –methoprene/amitraz/fipronil against *Rhipicephalus sanguineus* and *Ctenocephalides felis felis* on dogs. Parasit Vectors.

[28] Dryden MW, Payne PA, Smith V, Hostetler J (2006). Efficacy of imidacloprid (8.8 % w/w) plus permethrin (44 % w/w) spot-on topical solution against *Amblyomma americanum* infesting dogs using a natural tick exposure model. Vet Ther.

[29] Dumont P, Chester TS, Gale B, Soll M, Fourie JJ, Beugnet F (2015). Acaricidal efficacy of a new combination of fipronil and permethrin against *Ixodes ricinus* and *Rhipicephalus sanguineus* ticks. Parasit Vectors.

[30] Bonneau S, Reymond N, Gupta S, Navarro C (2015). Efficacy of a fixed combination of permethrin 54.5% and fipronil 6.1% (Effitix®) in dogs experimentally infested with *Ixodes ricinus*. Parasit Vectors.

[31] Dryden MW, Payne PA, Smith V, Hostetler J (2006). Evaluation of an imidacloprid (8.8 % w/w)-permethrin (44.0 % w/w) topical spot-on and a fipronil (9.8 % w/w)-(s)-methoprene (8.8 % w/w) topical spot-on to repel, prevent attachment, and kill adult *Ixodes scapularis* and *Amblyomma americanum* ticks on dogs. Vet Ther.

[32] Dryden MW, Payne PA, Smith V, Hostetler J (2006). Evaluation of an imidacloprid (8.8 % w/w)-permethrin (44.0 % w/w) topical spot-on and a fipronil (9.8 % w/w)-(s)-methoprene (8.8 % w/w) topical spot-on to repel, prevent attachment, and kill adult *Rhipicephalus sanguineus* and *Dermacentor variabilis* ticks on dogs. Vet Ther.

[33] Dumont P, Fourie JJ, Soll M, Beugnet F (2015). Repellency, prevention of attachment and acaricidal efficacy of a new combination of fipronil and permethrin against the main vector of canine babesiosis in Europe, *Dermacentor reticulatus* ticks. Parasit Vectors.

[34] Buczek A, Bartosik K, Kuczyński P (2014). Sensitivity to permethrin in a *Dermacentor reticulatus* population from eastern Poland in laboratory study. Parasit Vectors.

[35] Buczek A, Lachowska-Kotowska P, Bartosik K (2015). The effect of synthetic pyrethroids on the attachment and host-feeding behaviour in *Dermacentor reticulatus* females (Ixodida: Amblyommidae). Parasit Vectors.

[36] Lüssenhop J, Bäumer W, Kietzmann M, Schnieder T, Wolken S (2011). Dynamics of distribution and efficacy of different spot-on permethrin formulations in dogs artificially infested with *Dermacentor reticulatus*. Parasit Vectors.

[37] Dryden MW, Payne PA, McBride A, Mailen S, Smith V, Carithers D (2008). Efficacy of fipronil (9.8 % w/w) + (s)-methoprene (8.8 % w/w) and imidacloprid (8.8 % w/w) + permethrin (44 % w/w) against *Dermacentor variabilis* (American dog tick) on dogs. Vet Ther.

[38] Endris RG, Hair JA, Anderson G, Rose WB, Disch D, Meyer JA (2003). Efficacy of two 65 % permethrin spot-on formulations against induced infestations of *Ctenocephalides felis* (Insecta: Siphonaptera) and *Amblyomma americanum* (Acari: Ixodidae) on beagles. Vet Ther.

[39] Nicholson WL, Allen KE, McQuiston JH, Breitschwerdt EB, Little SE (2010). The increasing recognition of rickettsial pathogens in dogs and people. Trends Parasitol.

[40] Little SE. Changing paradigms in understanding transmission of canine tick-borne diseases: the role of interrupted feeding and intrastadial transmission. Proceedings of the 2nd Symposium of the CVBD World Forum. Mazara del Vallo: Bayer HealthCare AG, Leverkusen, Germany; 2007. p. 30–35.

[41] Fourie JJ, Stanneck D, Luus HG, Beugnet F, Wijnveld M, Jongejan F (2013). Transmission of *Ehrlichia canis* by *Rhipicephalus sanguineus* ticks feeding on dogs and on artificial membranes. Vet Parasitol.

[42] Hellmann K, Knoppe T, Krieger K, Stanneck D (2003). European multicenter field trial on the efficacy and safety of a topical formulation of imidacloprid and permethrin (Advantix) in dogs naturally infested with ticks and/or fleas. Parasitol Res.

[43] UK DEFRA. Summary of product characteristics, Advantix Spot-on solution for dogs over 25 kg. 2015. Available from: URL: www.vmd.defra.gov.uk/productinformationdatabase/spc_documents/spc_151145.doc. Accessed 1 June 2016.

[44] Varloud M, Hodgkins E (2015). Five-month comparative efficacy evaluation of three ectoparasiticides against adult cat fleas (*Ctenocephalides felis*), flea egg hatch and emergence, and adult brown dog ticks (*Rhipicephalus sanguineus* s.l.) on dogs housed outdoors. Parasitol Res.

[45] Fourie JJ, Beugnet F, Ollagnier C, Pollmeier MG (2011). Study of the sustained speed of kill of the combination of fipronil/amitraz/(S)-methoprene and the combination of imidacloprid/permethrin against *Dermacentor reticulatus*, the European dog tick. Parasite.

[46] Otranto D, Paradies P, Testini G, Latrofa MS, Weigl S, Cantacessi C (2008). Application of 10 % imidacloprid/50 % permethrin to prevent *Ehrlichia canis* exposure in dogs under natural conditions. Vet Parasitol.

[47] Otranto D, de Caprariis D, Lia RP, Tarallo V, Lorusso V, Testini G (2010). Prevention of endemic canine vector-borne diseases using imidacloprid 10 % and permethrin 50 % in young dogs: a longitudinal field study. Vet Parasitol.

[48] Fourie JJ, Luus HG, Stanneck D, Jongejan F (2013). The efficacy of Advantix® to prevent transmission of *Ehrlichia canis* to dogs by *Rhipicephalus sanguineus* ticks. Parasite.

[49] Navarro C, Reymond N, Fourie J, Hellmann K, Bonneau S (2015). Prevention of *Babesia canis* in dogs: efficacy of a fixed combination of permethrin and fipronil (Effitix®) using an experimental transmission blocking model with infected *Dermacentor reticulatus* ticks. Parasit Vectors.

[50] Blagburn BL, Spencer JA, Billeter SA, Drazenovich NL, Butler JM, Land TM (2004). Use of imidacloprid-permethrin to prevent transmission of *Anaplasma phagocytophilum* from naturally infected *Ixodes scapularis* ticks to dogs. Vet Ther.

[51] Spencer JA, Butler JM, Stafford KC, Pough MB, Levy SA, Bledsoe DL (2003). Evaluation of permethrin and imidacloprid for prevention of *Borrelia burgdorferi* transmission from blacklegged ticks (*Ixodes scapularis*) to *Borrelia burgdorferi*-free dogs. Parasitol Res.

[52] Cardoso L (2015). Fipronil and permethrin combination: a novel ectoparasiticide for dogs. Parasit Vectors.

[53] McMahon C, Kröber T, Guerin PM (2003). In vitro assays for repellents and deterrents for ticks: differing effects of products when tested with attractant or arrested stimuli. Med Vet Entomol.

[54] Achee N, Masuoka P, Smith P, Martin N, Chareonviryiphap T, Polsomboon S (2012). Identifying the effective concentration for spatial repellency of the dengue vector *Aedes aegypti*. Parasit Vectors.

[55] Achee NL, Sardelis MR, Dusfour I, Chauhan KR, Grieco JP (2009). Characterization of spatial repellent, contact irritant and toxic chemical actions of standard vector control compounds. J Am Mosquito Cont Assoc.

[56] Kröber T, Bourquin M, Guerin PM (2013). A standardised in vivo and in vitro test method for evaluating tick repellents. Pest Biochem Phys.

[57] Halos L, Baneth G, Beugnet F, Bowman AS, Chomel B, Farkas R (2012). Defining the concept of “tick repellency” in veterinary medicine. Parasitology.

[58] Liénard E, Bouhsira E, Jacquiet P, Warin S, Kaltsatos V, Franc M (2013). Efficacy of dinotefuran, permethrin and pyriproxyfen combination spot-on on dogs against *Phlebotomus perniciosus* and *Ctenocephalides canis*. Parasitol Res.

[59] Varloud M, Fourie JJ, Blagburn BL, Deflandre A (2015). Expellency, anti-feeding and speed of kill of a dinotefuran-permethrin-pyriproxyfen spot-on (Vectra®3D) in dogs weekly challenged with adult fleas (*Ctenocephalides felis*) for 1 month - comparison to a spinosad tablet (Comfortis®). Parasit Res.

[60] Beugnet F, Soll M, Bouhsira E, Franc M (2015). Sustained speed of kill and repellency of a novel combination of fipronil and permethrin against *Ctenocephalides canis* flea infestations in dogs. Parasite Vectors.

[61] Fankhauser B, Dumont P, Halos L, Hunter JS, Kunkle B, Everett WR (2015). Efficacy of a new combination of fipronil and permethrin against *Ctenocephalides felis* flea infestation in dogs. Parasite Vectors.

[62] Dryden MW (2009). Flea and tick control in the 21st century, challenges and opportunities. Vet Dermatol.

[63] Franc M, Cadiergues MC (1998). Antifeeding effect of several insecticidal formulations against *Ctenocephalides felis* on cats. Parasite.

[64] Ozoe Y, Asahi M, Ozoe F, Nakahira K, Mita T (2010). The antiparasitic isoxazoline A1443 is a potent blocker of insect ligand-gated chloride channels. Biochem Biophys Res Commun.

[65] Gassel M, Wolf C, Noack S, Williams H, Ilg T (2014). The novel isoxazoline ectoparasiticide fluralaner: selective inhibition of arthropod γ-aminobutyric acid- and l-glutamate-gated chloride channels and insecticidal/acaricidal activity. Insect Biochem and Mol Biol.

[66] Asahi M, Kobayashi M, Matsui H, Nakahira K (2015). Differential mechanisms of action of the novel γ-aminobutyric acid receptor antagonist ectoparasiticides fluralaner (A1443) and fipronil. Pest Manag Sci.

[67] Kilp S, Ramirez D, Allan MJ, Roepke RK, Nuernberger MC (2014). Pharmacokinetics of fluralaner in dogs following a single oral or intravenous administration. Parasite Vectors.

[68] García-Reynaga P, Zhao C, Sarpong R, Casida JE (2013). New GABA/glutamate receptor target for [3H] isoxazoline insecticide. Chem Res in Toxicol.

[69] Zhao C, Casida JE (2014). Insect γ-aminobutyric acid receptors and isoxazoline insecticides: toxicological profiles relative to the binding sites of [3H] fluralaner,[3H]-4′-ethynyl-4-n-propylbicycloorthobenzoate, and [3H] avermectin. J Agric Food Chem.

[70] Walther FM, Allan MJ, Roepke RK (2014). Nuernberger MC Safety of fluralaner chewable tablets (Bravecto™), a novel systemic antiparasitic drug, in dogs after oral administration. Parasit Vectors.

[71] Walther FM, Paul AJ, Allan MJ, Roepke RK, Nuernberger MC (2014). Safety of fluralaner, a novel systemic antiparasitic drug, in MDR1(−/−) Collies after oral administration. Parasit Vectors.

[72] Kilp S, Ramirez D, Allan MJ, Roepke RKA (2016). Comparative pharmacokinetics of fluralaner in dogs and cats following single topical or intravenous administration. Parasit Vectors.

[73] Taenzler J, Wengenmayer C, Williams H, Fourie J, Zschiesche E, Roepke RK (2014). Onset of activity of fluralaner (BRAVECTO™) against *Ctenocephalides felis* on dogs. Parasit Vectors.

[74] Walther FM, Allan MJ, Roepke RK, Nuernberger MC (2014). The effect of food on the pharmacokinetics of oral fluralaner in dogs. Parasit Vectors.

[75] Walther FM, Fisara P, Allan MJ, Roepke RK, Nuernberger MC (2014). Safety of concurrent treatment of dogs with fluralaner (Bravecto™) and milbemycin oxime - praziquantel. Parasit Vectors.

[76] Walther FM, Fisara P, Allan MJ, Roepke RK, Nuernberger MC (2014). Safety of the concurrent treatment of dogs with Bravecto (fluralaner) and Scalibor protectorband (deltamethrin). Parasit Vectors.

[77] Walther FM, Allan MJ, Roepke RK (2015). Plasma pharmacokinetic profile of fluralaner (Bravecto™) and ivermectin following concurrent administration to dogs. Parasit Vectors.

[78] European Commission. Bravecto EPAR summary for the public. 2016. Available from: URL: www.ema.europa.eu/ema/index.jsp?curl=pages/medicines/veterinary/medicines/002526/vet_med_000285.jsp&mid=WC0b01ac058001fa1c. Accessed 1 June 2016.

[79] Ghiasuddin SM, Kawauchi S, Matsumura F, Doherty JD (1982). Role of phospholipids in the inhibitory action of DDT and permethrin on the nerve ATPase of lobster, *Homarus americanus*. Biochem Pharmacol.

[80] Lawrence LJ, Gee KW, Yamamura HI (1984). Benzodiazepine anticonvulsant action: gamma-aminobutyric acid-dependent modulation of the chloride ionophore. Biochem Biophys Res Commun.

[81] Rohdich N, Roepke RK, Zschiesche E (2014). A randomized, blinded, controlled and multi-centered field study comparing the efficacy and safety of Bravecto™ (fluralaner) against Frontline™ (fipronil) in flea- and tick-infested dogs. Parasit Vectors.

[82] European Commission. Community register of veterinary medicinal products, Product information Bravecto Spot-On: Annex 1 Summary of product characteristics. 2016. Available from: URL: ec.europa.eu/health/documents/community-register/html/v158.htm. Accessed 1 June 2016.

[83] Taenzler J, Gale B, Zschiesche E, Roepke RKA, Heckeroth AR (2016). The effect of water and shampooing on the efficacy of Bravecto spot-on solution (fluralaner) against *Ixodes ricinus* and *Ctenocephalides felis* infestations in dogs. Parasit Vectors.

[84] Fisara P, Webster M (2015). A randomized controlled trial of the efficacy of orally administered fluralaner (Bravecto™) against induced *Ixodes holocyclus* (Australian paralysis tick) infestations on dogs. Parasit Vectors.

[85] Williams H, Zoller H, Roepke RK, Zschiesche E, Heckeroth AR (2015). Fluralaner activity against life stages of ticks using *Rhipicephalus sanguineus* and *Ornithodoros moubata* IN in vitro contact and feeding assays. Parasit Vectors.

[86] Williams H, Demeler J, Taenzler J, Roepke RKA, Zschiesche E, Heckeroth AR (2015). A quantitative evaluation of the extent of fluralaner uptake by ticks (*Ixodes ricinus*, *Ixodes scapularis*) in fluralaner (Bravecto™) treated vs. untreated dogs using the parameters tick weight and coxal index. Parasit Vectors.

[87] Wengenmayer C, Williams H, Zschiesche E, Moritz A, Langenstein J, Roepke RK, Heckeroth AR (2014). The speed of kill of fluralaner (Bravecto™) against *Ixodes ricinus* ticks on dogs. Parasit Vectors.

[88] Beugnet F, Liebenberg J, Halos L (2015). Comparative speed of efficacy against *Ctenocephalides felis* of two oral treatments for dogs containing either afoxolaner or fluralaner. Vet Parasitol.

[89] Beugnet F, Liebenberg J, Halos L (2015). Comparative efficacy of two oral treatments for dogs containing either afoxolaner or fluralaner against *Rhipicephalus sanguineus* sensu lato and *Dermacentor reticulatus*. Vet Parasitol.

[90] Taenzler J, Liebenberg J, Roepke RKA, Heckeroth AR (2015). Prevention of transmission of *Babesia canis* by *Dermacentor reticulatus* ticks to dogs treated orally with fluralaner chewable tablets (Bravecto™). Parasit Vectors.

[91] Dryden MW, Smith V, Bennett T (2015). Efficacy of fluralaner flavored chews (Bravecto®) administered to dogs against the adult cat flea, *Ctenocephalides felis felis* and egg production. Parasit Vectors.

[92] Williams H, Young DR, Qureshi T, Zoller H, Heckeroth AR (2014). Fluralaner, a novel isoxazoline, prevents flea (*Ctenocephalides felis*) reproduction in vitro and in a simulated home environment. Parasit Vectors.

[93] Meadows C, Guerino F, Sun F (2014). A randomized, blinded, controlled USA field study to assess the use of fluralaner tablets in controlling canine flea infestations. Parasit Vectors.

[94] Coles TB, Dryden MW (2014). Insecticide/acaricide resistance in fleas and ticks infesting dogs and cats. Parasit Vectors.

